# Life threatening intracerebral haemorrhage following saw- scaled viper (*Echis carinatus*) envenoming-authenticated case report from Sri Lanka

**DOI:** 10.1186/1471-227X-13-5

**Published:** 2013-04-08

**Authors:** Chathuranga Lakmal Fonseka, Vijayabala Jeevagan, Christeine Ariaranee Gnanathasan

**Affiliations:** 1 National Hospital Sri Lanka, University medical unit, Colombo, Sri Lanka; 2 National Hospital, University medical unit, Colombo, Sri Lanka; 3 Faculty of Medicine, University of Colombo, Colombo, Sri Lanka; 4 National Hospital of Sri Lanka, Regent Street, Colombo 8, Sri Lanka

## Abstract

**Background:**

*Echis carinatus* (Saw scaled viper {SSV}) is a venomous snake found in the parts of Middle East and Central Asia. SSV envenoming is characterized by local swelling and coagulopathy. Various bleeding manifestations are commonly seen with SSV envenoming. In contrast to other part of Asia, saw scale viper envenoming has not been reported to cause life threatening haemorrhagic manifestations in Sri Lanka.

**Case presentation:**

We report a 19 years old healthy boy who developed massive left temporo-parietal intra cerebral haemorrhage following *Echis carinatus* (Saw scaled viper) bite in Sri Lanka.

**Conclusion:**

Although subspecies of SSV in Sri Lanka is regarded as a ‘non lethal venomous snake’, the occurrence of rare potentially fatal complications such as intracerebral haemorrhage should be considered in their management. This case report is intended to bring the awareness of this fatal complication of SSV envenoming in Sri Lanka.

## Background

*Echis carinatus* (Saw scaled viper {SSV}) is a venomous snake found in the parts of Middle East and Central Asia [1]. In Sri Lanka SSV is found in the dry coastal plains of northern, north-western and eastern provinces [2,3]. SSV envenoming is characterized by local swelling and coagulopathy. Various bleeding manifestations are commonly seen with SSV envenoming. Common bleeding manifestations include gingival bleeding, haematuria, epistaxis, haemoptysis and haematemesis. In a case series involving 48 SSV bite victims, 71% had coagulopathy as evidence by 20 min WBCT. Spontaneous bleeding occurred in 29% and majority of them had either haematuria or haematemesis [4]. All the published case series of SSV bite in Sri Lanka failed to report any life threatening bleeding manifestations such as retoperitoneal, plero-pericardial or intracranial bleeding [4-6]. Fatalities due to SSV envenoming have not been reported in Sri Lanka. Therefore, in contrast to other countries SSV envenoming in Sri Lanka is regarded as nonlethal and moderate venomous.

Here we report a 19 year old healthy boy who developed left massive temporo-parietal intra cerebral hemorrhage following SSV envenoming. Our case is the first case of intracerebral bleeding following saw- scaled viper envenoming in Sri Lanka. Pathophysiology of venom induced consumptive coagulopathy is discussed in order to understand the resultant coagulopathy from this envenoming.

## Case presentation

**Figure 1:**
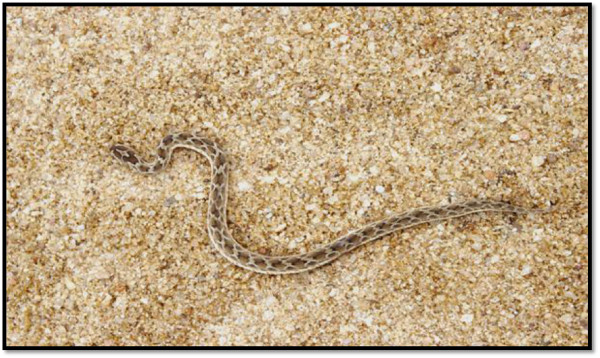
Example of a live saw- scaled viper.

A 19 years old healthy boy was bitten by a snake in his left foot while he was walking in his garden. The killed snake was brought to the hospital and identified as *Echis carinatus* (Figure 1) by the attending medical officer and one of the authors (CAG). On admission to the local hospital, there was mild local bleeding at the bite site, but there was no clinical evidence of systemic envenoming. Three hours after the bite he had developed progressive headache and his blood was found to be incoagulable by the 20 minutes Whole blood clotting test (20WBCT). He was treated immediately with 10 vials of polyvalent antivenom serum (AVS) {Vins Bioproduct}, raised against Indian *Daboia russelii*, *Echis carinatus*, *Naja naja* and *Bungarus caeruleus* venoms, each vial was dissolved in 10 ml of sterile water and diluted with 200 ml of normal saline to a total volume of 300 ml and was infused intravenously over an hour to restore the coagulability. Despite of restoration of coagulability, the headache persisted throughout without any demonstrable neurological deficit.

Following day, he had developed right sided complete ptosis with fixed dilated pupil. On detection of these neurological features the boy was immediately transferred to the University Medical unit, National Hospital of Sri Lanka.

**Figure 2:**
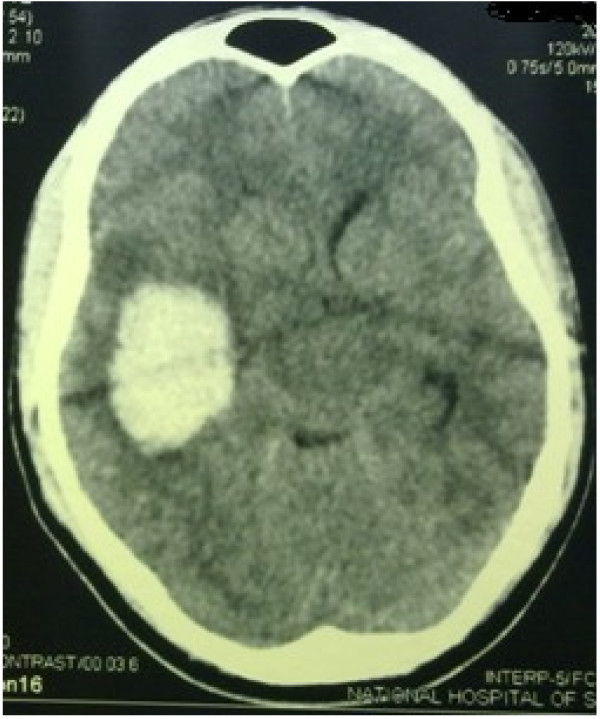
Non-contrast CT brain showing a massive left temporo-parietal region intra-cerebral haemorrhage.

On admission to our unit, his Glasgow Coma Scale (GCS) was 13/15. Cranial nerve examination confirmed right sided complete ptosis with fixed dilated pupil. Fundoscopic examination failed to revealed papilloedema. Upper and lower limbs were neurologically normal. His blood pressure was 130/80 mm Hg with pulse rate of 66 beats/min and respiratory rate was 14/min. There was no evidence of external bleeding. The blood was coagulable by 20WBCT. The urgent non-contrast CT brain showed a massive left temporo-parietal region intra-cerebral haemorrhage with intra-ventricular extension (Figure 2). His vital parameters and GCS were monitored regularly.

Laboratory investigations revealed a haemoglobin of 13.2 g/dl, total leukocyte count of 13,500, with 80% neutrophils, platelet count of 178,000/mm3. Coagulation profile showed prothrombin time (PT) 18 seconds (control 12 s) with INR of 1.44 thromboplastin time with kaolin (PTTK) was 38 seconds (control-35 s) and fibrinogen degradation products was 2.72 mg/l (Normal-<0.20 mg/l). The biochemical investigations including blood urea, serum creatinine, plasma glucose, serum bilirubin and transaminases were with in normal limit.

An urgent Digital Subtraction Angiography (DSA) of the intracranial vessel was performed and which was normal excluding the possibility of ruptured aneurysm or an arterio-venous malformation. Since his clinical parameters were improving, he was managed conservatively without any neurosurgical intervention. He was discharged home a week later in good health without any neurological deficit.

## Discussion and conclusion

Saw- scaled viper venom is a highly complex mixture of a variety of biological substances including protein and non protein toxins [7,8]. Multiple mechanisms have been suggested for coagulopathy following SSV envenoming. The most common coagulopathy associated with snake bite envenoming is venom induced consumptive coagulopathy(VICC) [9].

A VICC result from the activation of the coagualation pathway in varied points by procoagulant toxins. SSV venom contains two metalloproteinases that are prothrombin activators namely ecarin and carinactivase [7,8]. Activation of prothrombin by these factors result in consumptive coagulopathy with variable deficiency in fibrinogen, factor V and factor VII [9]. Simultaneous injury to the blood vessel integrity increases the risk of bleeding. The venom also contains factor X activator and many other compounds which increases its capacity to cause coagulopathy such as platelet aggregation inhibitors [7-9]. VICC and direct endothelial injury due to haemorrhagin in the venom might be responsible for the near fatal intracerebral haemorrhage in our patient. VICC is characterized by prolonged 20WBCT, PT and PTTK and a marked increase in fibrinogen degradation products.

Personal communication with many practicing physicians in the Island revealed that there is a wide variation in the treatment methods used in SSV envenoming. Because of the belief, that the Sri Lankan subspecies of SSV is never fatal to man, AVS is not used by all. Although subspecies of SSV in Sri Lanka is regarded as a ‘non lethal venomous snake’, the occurrence of rare potentially fatal complications such as intracerebral haemorrhage should be considered in their management. AVS should be administered promptly if features of systemic envenoming are present. In our patient neurological deficit was progressive despite the prompt use of AVS. It indicates either AVS was less effective in preventing progression of intracerebral haemorrhage or inadequate dose of AVS was used. It is essential to evaluate the effectiveness of AVS against specific subspecies of SSV in Sri Lanka, so that AVS should be used if it is beneficial only. The use of Fresh Frozen Plasma (FFP) in VICC remains controversial and only few studies investigating this issue. In a study included 167 cases of VICC following snake bite envenoming in Australia, showed AVS was ineffective in restoration of coagulopathy [10]. Interestingly FFP replacement was associated with faster recovery and reduced the risk of bleeding [10]. These findings should be confirmed in well designed randomized controlled trails in Sri Lankan ***Echis carinatus***, before making any conclusions. This case report is intended to bring the awareness of this fatal complication of SSV envenoming in Sri Lanka.

## Consent

Written informed consent was obtained from the patient for publication of this Case report and any accompanying images. A copy of the written consent is available for review by the Editor of this journal.

## Competing interests

The authors declare that they have no competing interests.

## Authors’ contributions

VJ and CLF carried out the literature search and drafted the manuscript; CAG did the critical revision for important intellectual content in the manuscript and given the final approval of the version to be published; all the authors read and approved the final manuscript.

## Pre-publication history

The pre-publication history for this paper can be accessed here:

http://www.biomedcentral.com/1471-227X/13/5/prepub
